# The Impact of Cognitive Behavioral Counseling in Promoting Self-Healing of Irritable Bowel Syndrome: Longitudinal Study

**DOI:** 10.1192/j.eurpsy.2023.1031

**Published:** 2023-07-19

**Authors:** N. S. G. Abdelrasheed, M. A. RearOmer, A. A. Almaashani, M. A. Khalaf

**Affiliations:** 1 Department of Education - Psychological Counseling Specialization, Dhofar University; 2 Ministry of Education, Ministry of Education; 3 Chair of Dr. Ahmed Almaashani center for Psychological and family counseling, Dr. Ahmed Almaashani center for Psychological and family counseling, Salalah; 4 Department of Psychology, Sultan Qaboos University, Muscat, Oman; 5 Educational Psychology, Minia University, Minia, Egypt

## Abstract

**Introduction:**

Psychophysical diseases can be cured without medical intervention, and this is the so-called self-healing. Self-healing is the process of recovery from emotional ill-health, but self-healing can also include accompanying physical health issues. Cognitive-behavioral therapy is one of the effective methods that help the individual to activate their role in self-healing and controlling thoughts and lifestyle. Being therapeutically effective in previous literature and less expensive than medication, CBT can be utilized by psychiatry practitioners.

**Objectives:**

The current study aimed at investigating the effects of a cognitive behavioral therapy-based program to promote self-healing of patients with irritable bowel syndrome (IBS). Additionally, it explores the continuity of the proposed program effectiveness throughout six months.

**Methods:**

The quasi-experimental method (one group design) was adopted Participants were 4 patients (ages between 29-34 years) were purposively selected since they were suffering from irritable bowel syndrome (IBS) for (4-11) years based on the diagnosis conducted by Gastroenterology Clinic specialists at Sultan Qaboos Hospital in Salalah. The fifteen sessions of the therapeutic intervention lasted for five weeks. No medications were taken during the intervention and the follow-up period.

**Results:**

Results indicated the effectiveness of the intervention in promoting self-healing of the irritable bowel syndrome (IBS) and a decrease in the symptoms of the irritable bowel in the medical examination after intervention as shown in the significant differences between time 1 and time 2 assessment while no significant difference was detected between time 2 and time 3 assessment (follow-up). A significant decrease in the medical symptoms of IBS (85% improvement rate).

**Conclusions:**

Non-pharmacological psychotherapy is beneficial with patients with psychosomatic disorders as it can be used effectively to improve self-healing.

**Disclosure of Interest:**

None Declared

